# Human Bone-Marrow-Derived Stem-Cell-Seeded 3D Chitosan–Gelatin–Genipin Scaffolds Show Enhanced Extracellular Matrix Mineralization When Cultured under a Perfusion Flow in Osteogenic Medium

**DOI:** 10.3390/ma16175898

**Published:** 2023-08-29

**Authors:** Gabriele Boretti, Emanuele Giordano, Mariana Ionita, George Mihail Vlasceanu, Ólafur Eysteinn Sigurjónsson, Paolo Gargiulo, Joseph Lovecchio

**Affiliations:** 1 School of Science and Engineering, Reykjavík University, 102 Reykjavík, Iceland; gabriele23@ru.is (G.B.); oes@ru.is (Ó.E.S.); paolo@ru.is (P.G.); josephl@ru.is (J.L.); 2 Laboratory of Cellular and Molecular Engineering “Silvio Cavalcanti”, Department of Electrical, Electronic and Information Engineering “Guglielmo Marconi” (DEI), University of Bologna, 47522 Cesena, FC, Italy; 3 Advanced Research Center on Electronic Systems (ARCES), University of Bologna, 40126 Bologna, BO, Italy; 4 Faculty of Medical Engineering, University Politehnica of Bucharest, 060042 Bucharest, Romania; mariana.ionita@polimi.it (M.I.); vlasceanu.georgemihail@yahoo.ro (G.M.V.); 5 Advanced Polymer Materials Group, University Politehnica of Bucharest, 060042 Bucharest, Romania; 6 eBio-Hub Research Centre, University Politehnica of Bucharest-Campus, 060042 Bucharest, Romania; 7 The Blood Bank, Landspitali, The National University Hospital of Iceland, 105 Reykjavík, Iceland; 8 Institute of Biomedical and Neural Engineering, Reykjavik University, 102 Reykjavík, Iceland

**Keywords:** biomaterials, bioreactors, chitosan, gelatin, graphene oxide, human bone-marrow-derived mesenchymal stem cells, regenerative medicine, scaffolds, tissue engineering

## Abstract

Tissue-engineered bone tissue grafts are a promising alternative to the more conventional use of natural donor bone grafts. However, choosing an appropriate biomaterial/scaffold to sustain cell survival, proliferation, and differentiation in a 3D environment remains one of the most critical issues in this domain. Recently, chitosan/gelatin/genipin (CGG) hybrid scaffolds have been proven as a more suitable environment to induce osteogenic commitment in undifferentiated cells when doped with graphene oxide (GO). Some concern is, however, raised towards the use of graphene and graphene-related material in medical applications. The purpose of this work was thus to check if the osteogenic potential of CGG scaffolds without added GO could be increased by improving the medium diffusion in a 3D culture of differentiating cells. To this aim, the level of extracellular matrix (ECM) mineralization was evaluated in human bone-marrow-derived stem cell (hBMSC)-seeded 3D CGG scaffolds upon culture under a perfusion flow in a dedicated custom-made bioreactor system. One week after initiating dynamic culture, histological/histochemical evaluations of CGG scaffolds were carried out to analyze the early osteogenic commitment of the culture. The analyses show the enhanced ECM mineralization of the 3D perfused culture compared to the static counterpart. The results of this investigation reveal a new perspective on more efficient clinical applications of CGG scaffolds without added GO.

## 1. Introduction

Tissue-engineered bone tissue grafts are viewed as a promising alternative to the conventional use of natural donor bone grafts in terms of protocol standardization, but their effective translation into clinical practice is still a challenge [1,2,3,4]. In particular, choosing an appropriate biomaterial/scaffold to sustain cell survival, proliferation and differentiation in a 3D environment remains one of the most critical aspects to approach [5,6]. Several natural or synthetic biomaterials have been used to this aim [7,8,9,10,11,12,13,14]. In this respect, we recently proposed chitosan/gelatin/genipin (CGG) hybrid scaffolds doped/reinforced with graphene oxide (GO) as a promising biomaterial/scaffold with osteogenic potential [15]. Some concern was however raised towards the use of graphene and graphene-related material in medical applications, requiring further testing and full understanding of their behavior in biological systems [16,17]. While the results of such studies are ongoing, we here thus propose to evaluate the osteogenic potential of CGG scaffolds [18,19] without added GO, by including a perfusion flow into the differentiation-inducing protocol. In fact, it is well established that a dynamic environment can improve diffusion gradients, especially within tridimensional tissue constructs, and thus enhance their functional behavior. Indeed, several studies [20,21,22,23,24,25,26] demonstrated that the use of bioreactor systems to this aim can improve cell phenotype commitment. Thus, here, human bone-marrow-derived stem cell (hBMSC)-seeded 3D CGG scaffolds have been cultured under a perfusion flow and the extent of the early osteogenic process [27,28,29,30,31,32] has been evaluated as the level of extracellular matrix (ECM) mineralization was measured. An increased ECM mineralization was found in 3D CGG scaffolds cultured in dynamic conditions compared to static conditions. The results of this investigation reveal a new perspective on potential clinical applications of CGG scaffolds without added GO.

## 2. Materials and Methods

### 2.1. Three-Dimensional Chitosan–Gelatin–Genipin Scaffolds

Crab-shell-derived medium-molecular-weight chitosan with a 75–85% deacetylation degree, cold water fish gelatin, genipin (purity > 98%—HPLC grade), and acetic acid (>99.7%) were purchased from Sigma Aldrich (St. Louis, MO, USA) and used without prior purification. The composites’ synthesis was carried out in double distilled water. Genipin crosslinked gelatin/chitosan blend scaffolds were prepared under identical conditions as previously reported [33]. Briefly, gelatin was dissolved in water (5% *w/v*) by thoroughly stirring at 50 °C for 1 h and mixed with a 1% wt. chitosan solution prepared in a mild acidic solution (1% *v/v*) by stirring overnight at 40 °C. For a total of 50 mL of solution, 8.33 mL of gelatin solution was homogenized with 41.67 mL chitosan solution. The crosslinking was carried out with genipin (1% *w/w* in water). Next, materials were frozen at −80 °C and freeze-dried (−55 °C).

### 2.2. Perfusion Bioreactor

A perfusion bioreactor system was used to generate a continuous perfusion flow in a range of 0.25 to 1 mL/min, aiming at obtaining a constant supply of nutrients and the removal of waste products to sustain the 3D cell culture [34,35,36]. The device consists of (i) a unibody plastic case (190 L, 240 W, and 90 H mm in dimension); (ii) two autoclavable WPM2-S1EACP peristaltic pumps (WELCO, Tokyo, Japan); (iii) an appropriate set of 3D printed culture plates. A control unit hosting an Arduino UNO, two EasyDriver stepper motor drivers (SparkFun Electronics, Boulder, CO, USA), and a PMB-12V35W1AA power supply (Delta Electronics, Taipei, Taiwan) was designed to guide the two peristaltic pumps. The control unit was connected via a USB cable to a laptop that allows easy tuning the perfusion flow via a graphical user interface (GUI). The main body of the device is intended to operate inside a standard cell culture incubator to maintain a physiological pH and temperature.

#### 2.2.1. Three-Dimensional-Printed Culture Plates

A set of cultures plates was designed and 3D printed using a Formlabs3 3D printer (Formlabs, Somerville, MA, USA). The culture plate layout was designed by Fusion360 CAD software (Autodesk, San Rafael, CA, USA) and the generated file was exported in a .stl format, imported into PreForm software (Formlabs, Somerville, MA, USA), and 3D printed. The plates were intended to operate independently to allow comparison of two different culture conditions. Holes (1.6 mm in diameter) were placed between adjacent wells to allow a medium flow. The final culture plate layout consists of two 6-well plates made from DentalLT photo-polymer resin (Formlabs, Somerville, MA, USA). This material is approved for biomedical applications. Culture plates were post-cured using FormWash and FormCure devices (Formlabs, Somerville, MA, USA) following the manufacturer’s guidelines.

#### 2.2.2. Graphical User Interface

A user-friendly GUI was implemented in Processing 3 (Processing Foundation, San Diego, CA, USA). This interface allows us to easily administer a perfusion flow rate at three different rates: 0.25, 0.5, and 1 mL/min. Moreover, each perfusion pump can be turned on and off independently. The direction of the rotation can also be set independently. The GUI can be exported to work both on Windows or Mac OS 3.5.4.

### 2.3. Human Mesenchymal Stem Cell Culture

Primary human bone-marrow-derived mesenchymal stem cells (hBMSCs) were purchased from Lonza Inc. (Allendale, NJ, USA) and used at passage #2 for the seeding onboard 3D scaffolds. A one-week culture followed, either within the bioreactor system or under conventional static conditions as a control. To this aim, the cells were initially expanded as a 2D monolayer in high glucose Dulbecco’s Modified Eagle Medium (DMEM) containing 0.1% penicillin/streptomycin, 0.1 mM non-essential amino acids, and 10% fetal bovine serum. After two passages, the hBMSCs were trypsinized and counted in a hemocytometer using Trypan Blue staining to evaluate the number of dead cells. All reagents were purchased from Life Technologies (Carlsbad, CA, USA).

### 2.4. Cell Culture

hBMSCs at passage #2 were seeded at a concentration of 85,000/50 μL onboard eight 3D chitosan–gelatin–genipin scaffolds (7 mm in height and 5 mm in diameter). Four scaffolds were cultured in either static control or dynamic culture conditions. A 1 mL/min perfusion flow was administered according to [37,38]. To avoid cell loss, scaffold seeding was performed in a 96-well plate, adding over the dry scaffold 50 μL cell solution. Then, 150 μL of medium was added after 2 h.

#### Osteogenic Differentiation

Scaffolds were moved into the 3D-printed culture plates 36 h after cell seeding.

**Figure 1:**
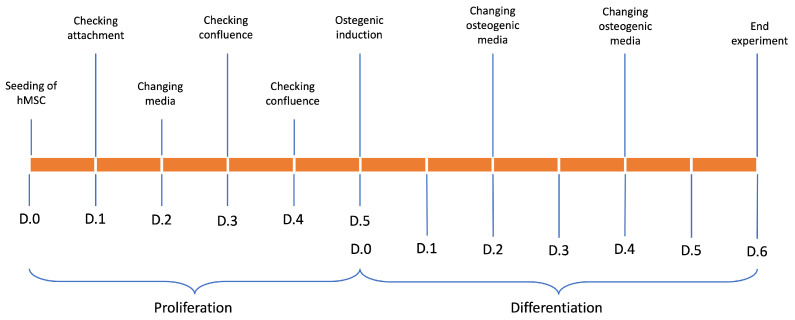
Timeline of experimental setting.

As shown in Figure 1, the experiment was carried out for eleven days. hMSCs seeded onboard chitosan–gelatin–genipin scaffolds were cultured for five days in 30 mL of proliferative medium per plate. Subsequently, for the next six days, early osteogenic differentiation was induced by adding ascorbic acid, bone morphogenic protein-2 (BMP2), and beta-glicerophosphate to the proliferative medium [39,40,41]. Half of the exhausted medium (15 mL) was changed every 48 h in both dynamic and static cultures.

### 2.5. Micro-Computer Tomography (μCT)

μCT scanning was performed using a SkyScan 1272 micro-computer (Bruker Corporation, Billerica, MA, USA) tomograph at room temperature by rotating the object in front of the source (voltage 45 kV, current 110 mA) for 180 degrees with a rotation step of 0.3 degrees; each frame resulted from an average of 4 projections per frame (550 ms/frame). The scanning resolution (image pixel size) was set at 15 μm for all printed samples. Tomograms were reconstructed from the raw data in Bruker NRecon software (Bruker Corporation, Billerica, MA, USA). Bruker CTAn software (Bruker Corporation, Billerica, MA, USA) was employed to analyze the tomograms and measure the morphological parameters of the printed objects (total porosity, pore/wall size distribution, etc.) and to generate the secondary color-coded dataset depicting pore size variations. All procedures were performed after thresholding (binarization; white pixels for solid sample and black pixels for pores) and despeckling (removal or residual scanning artifacts) and were based on the image pixel size for metric unit conversion.

### 2.6. Histochemical Assay and Image Analisys

Samples fixed in 4% paraformaldhyde and embedded in paraffin were cut into 5.0 μm thick sections. Samples were stained with hematoxylin and eosin (H&E) for morphological analyses according to [42], and with Alizarin Red S (ARS) to highlight calcium deposits according to [43], indicative of a mineralization process initiated by cells differentiating into an osteogenic phenotype. A quantitative analysis of ECM mineralization (i.e., calcium salt deposits) was performed on images acquired from ARS-stained chitosan–gelatin–genipin scaffold slices. Digital images were acquired in each experimental condition with a Eclipse TE 2000U (Nikon, Tokyo, Japan) optical inverted microscope mounting a 4× Nikon CFI Plan Fluor objective through a DS-5Mc digital camera (Nikon, Tokyo, Japan) operated under a DS-U1 controller connected to a Dell XPS desktop PC equipped with ImageJ (National Institute of Health, Bethesda, MD, USA) image analysis software (version 1.51.m9).

Calcium salt deposits were evaluated in ARS-stained sections using the maximum entropy threshold-based image segmentation method. The count and the average size of the calcium salt deposits, as well the total image area they covered, were calculated. Control (static culture) and dynamic (bioreactor system) data were compared.

## 3. Results and Discussion

Chitosan/gelatin/genipin (CGG) scaffolds, with an intrinsic characteristic of supporting osteogenic induction in stem cells, were made more efficient through the addition of graphene oxide (GO) [15,44]. Although the properties of this biomaterial have been tested, there is some degree of concern about hypothetical side effects [16]. For this reason, while the results of studies about this issue are pending, we evaluated if a dynamic culture of CGG scaffolds without added GO could increase their osteogenic potential, as heralded in previous work [45,46,47,48]. Thus, the in vitro osteogenic potential of the CGG scaffold was investigated in either conventional (i.e., static) or perfusion flow (i.e., dynamic) conditions. The degree of calcium salt deposition by the hBMSCs onboard the 3D scaffold was used as the end-point of the analysis.

### 3.1. Characterization of CGG Scaffolds

**Figure 2:**
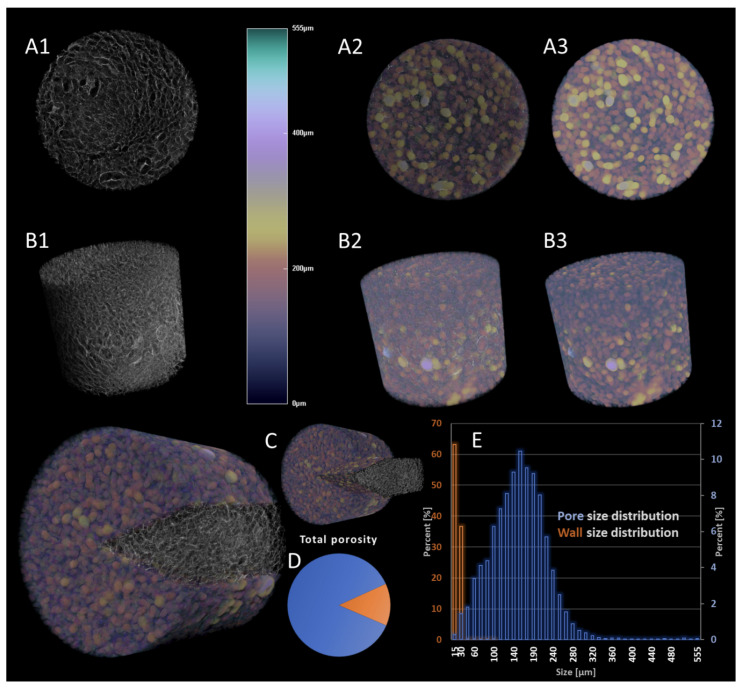
Morphological characterization of chitosan–gelatin–genipin scaffolds by μCT. (**A1**–**A3**) Cross-sectional morphology of the acellular freeze-dried sample; (**B1**–**B3**) overall view of the scanned sample; subset 1, morphology of the solid sample; subset 2, pore reconstruction overlapped to solid objects; subset 3, pore reconstruction and relative pore size according to the color legend. (**C**) Subdivision illustrates a dual view of the solid sample and overlapped pore dataset with the solid. (**D**,**E**) Plot of the pore size and wall thickness distribution.

Morphological characterization of the material before cell seeding (i.e., post-synthesis), presented in Figure 2, confirmed the suitability of the CGG scaffold to host the hBMSCs. In detail, qualitative and quantitative analyses were carried out by μCT. Figure 2 shows the 3D reconstruction of a sample scaffold (Figure 2(A1,B1)), as well as the color-coded secondary reconstruction of the pore size (Figure 2(A3,B3)) overlapped with the tomogram of the solid specimen (Figure 2(A2,B2,C)) for a better visualization and comprehension of its intricate pattern and interfaces. The solid sample exhibits a homogeneous architecture, whereby the wall thickness varies to a small extent (with a ratio of more than 99% below 40 μm), contributing to its identification as a consolidated and robust scaffolding material. However, the pores that are formed span to a broader extent (up to 555 μm), but in a rather balanced Gaussian distribution (Figure 2E). Despite its perceptible solidity, the measured porosity of the freeze-dried material achieves values of 86.8% (Figure 2D); the analysis of pore size domains depicts a great variability that is favorable for both cell lodging and post-adhesion cellular activity, as well as metabolite exchange, supporting these processes for viable tissue construct attainment. The very high incidence (77%) of pores within the range of 120–300 μm could support the employment of this material for transitional total porosity, as it is able to provide accessibility channels for cell nutrients. In addition, the distribution of small pores is extremely homogeneous within the sample (Figure 2, subsets 2, 3), contributing to the interconnected nature of the scaffolding template, a crucial prerequisite of effective materials for tissue regeneration. Furthermore, the surface area of the sample is paramount, especially in the incipient post-seeding stages of cell culture. The surface area of the scanned specimen was measured at 1.11 × 10^8^ μm^2^; however, more relevant for material characterization is the specimen-independent measurement of the object surface/volume ratio, which reached the value of 1.97 μm^−1^.

### 3.2. Matrix Mineralization upon Dynamic Culture

To overcome the diffusion constraints inherent to a 3D cell culture in a conventional static environment, a custom bioreactor system was used to generate a continuous perfusion flow to sustain cell growth.

**Figure 3:**
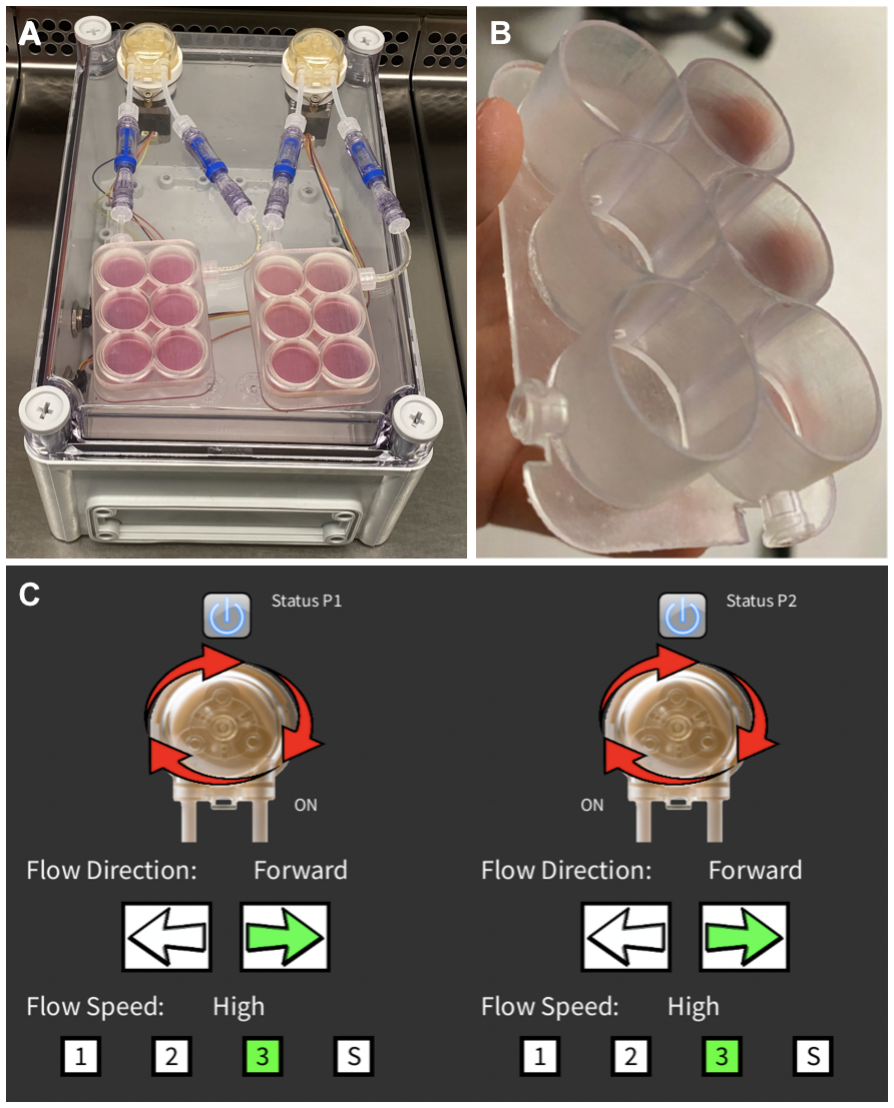
Perfusion bioreactor system overview. (**A**) Perfusion bioreactor; (**B**) Six-well plate; (**C**) Graphical user interface (GUI): peristaltic pumps (P1 and P2) status (ON/OFF), flow direction, and flow speed.

Building on our experience in designing and prototyping devices for dynamic culture [37,38,49], we here developed a dedicated tool (Figure 3A) consisting of a unibody plastic case (190 L, 240 W, and 90 H mm in dimensions) and two autoclavable peristaltic pumps acting on two distinct ad hoc culture plates (Figure 3B) and operated by an original graphical user interface (GUI, Figure 3C). This new layout consists of two six-well plates made from DentalLT photo-polymer resin (Formlabs, Somerville, MA, USA) and operated independently to allow comparison of two different culture conditions. In particular, holes (1.6 mm in diameter) were placed between adjacent wells to allow a medium flow.

**Figure 4:**
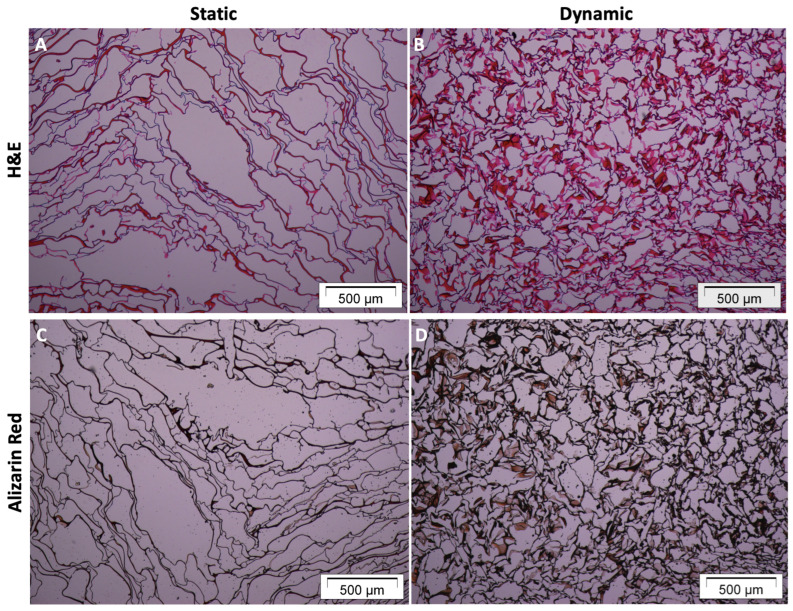
Histological analysis of the 3D chitosan–gelatin–genipin scaffold seeded with hBMSCs and subjected to six days of osteogenic induction. (**A**,**B**) Hematoxylin and eosin assay on scaffolds cultured in static (**A**) vs. dynamic (**B**) conditions; (**C**,**D**) Alizarin Red S assay on scaffolds cultured in static (**C**) vs. dynamic (**D**) conditions. Pictures acquired at 40× magnification, using Cell^A Software (version 2.5). Scale bar 500 μm.

Figure 4A,B shows the viability and distribution of the hBMSCs seeded in the 3D CGG scaffolds. Hematoxylin/eosin staining analysis highlighted a higher amount of cells (purple spots) in dynamic conditions (Figure 4B) compared to static conditions (Figure 4A). Figure 4C,D shows that 3D-perfused CGG scaffolds display enhanced levels of extracellular matrix (ECM) mineralization, suggesting an early osteogenic commitment. In fact, alizarin red S staining analysis highlighted a higher amount of calcium deposits (black spots) in dynamic conditions (Figure 4D) compared to static conditions (Figure 4C). For both analyses, a remodelling of the scaffold structure was observed when hBMSCs were cultured in dynamic conditions, resulting in thickening of the scaffold texture.

**Figure 5:**
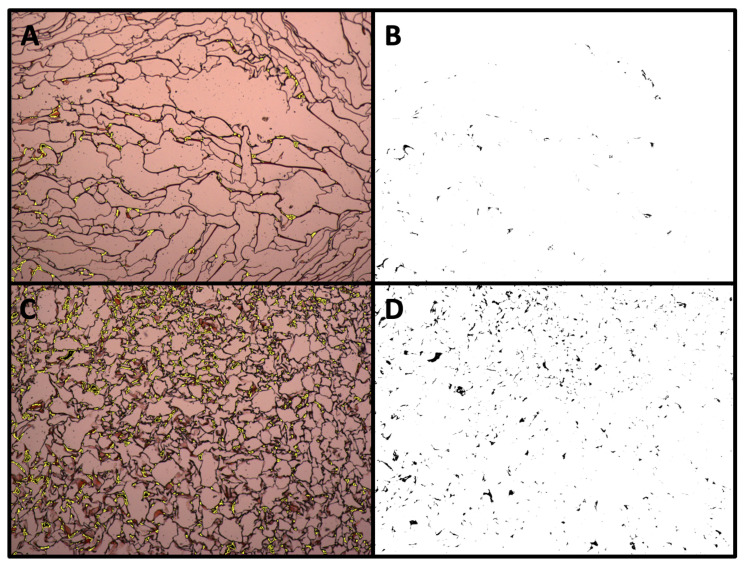
Quantification of calcium deposits using alizarin red S staining. (**A**) Static conditions; (**C**) dynamic conditions; (**B**,**D**) segmented areas of the calcium salt deposits.

A detailed quantification of the calcium salt deposit amount, carried out through the maximum entropy threshold-based image segmentation method, is presented in Figure 5. In particular, Figure 5B shows the segmented area referring to the calcium salt deposition over the 3D CGG scaffold fibers by the hBMSCs when cultured under static condition (Figure 5A). Figure 5D shows the segmented area referring to the calcium salt deposition over the 3D CGG scaffold fibers by the hBMSCs when cultured under dynamic conditions (Figure 5C).

The amount of scaffold area covered by the calcium salt deposits in both static and dynamic conditions is reported in Table 1. The results are reported as the count and average size of the calcium salts, as well as the total image area covered. Higher values were obtained for 3D CGG scaffolds cultured in dynamic conditions.

## 4. Conclusions

The purpose of this work was to improve the osteogenic potential of chitosan/gelatin/genipin (CGG) scaffolds without added graphene oxide (GO) by including a perfusion flow into the differentiating protocol of human bone-marrow-derived stem cells seeded onboard the scaffold. A histological analysis showed enhanced extracellular matrix mineralization under a perfusion flow in an osteogenic medium, suggesting an early osteogenic commitment. The results of this investigation reveal a new perspective on potential clinical applications of CGG scaffolds without added GO.

## Figures and Tables

**Table 1:** Amount of scaffold area covered by the calcium salts.

Slice	Count	Total Area	Average Size
ARS Static	892	0.610	6.837 × 10${}^{-4}$
ARS Dynamic	4754	6.730	0.001
